# Engaging Native American Caregivers in Youth-Focused Diabetes Prevention and Management

**DOI:** 10.5888/pcd15.170521

**Published:** 2018-06-21

**Authors:** Rachel Chambers, Summer Rosenstock, Melissa Walls, Anne Kenney, Marissa Begay, Kendrea Jackson, Leonela Nelson, Nicole Neault, Novalene Goklish, Dike Van De Mheen, Allison Barlow

**Affiliations:** 1 Center for American Indian Health, International Health Department, Johns Hopkins Bloomberg School of Public Health, Baltimore, Maryland; 2 Department of Family Medicine and Biobehavioral Health, University of Minnesota Medical School, Duluth Campus, Duluth, Minnesota; 3 Tilburg University, Scientific Centre for Care and Welfare (Tranzo), Tilburg, Netherlands

## Abstract

Native American youth aged 10 to 19 years are disproportionately affected by type 2 diabetes. Intergenerational programs may improve health in tribal communities. We evaluated Together on Diabetes, a diabetes prevention and management program, among 257 participating Native American youths with or at risk for type 2 diabetes and their adult caregivers. Feasibility, acceptability, and demographic data were collected from 226 adult caregivers. Data on physical measurements (weight, height, waist circumference) were collected from 37 of the caregivers. Results indicated that engaging adult caregivers was feasible, acceptable, and effective. Furthermore, a subset of adult caregivers reduced their body mass index (weight in kilograms divided by height in m^2^) significantly from the start to the end of the program, a 12 month period (*P* = .02). Findings suggest the feasibility of engaging adult caregivers in youth diabetes prevention programs.

## Objective

Native American youth are disproportionally affected by type 2 diabetes (hereinafter diabetes) (1). Because youth and their adult caregivers often share risk factors for diabetes (2), engaging caregivers in youth diabetes prevention programs may positively affect adults while increasing program effectiveness for youth. Intergenerational designs may also strengthen public health efforts and cost effectiveness — key considerations for low-resource Native American communities in which 1 in 6 adults had diabetes in 2015 (3). We explored data from adult caregivers enrolled in the Together on Diabetes (TOD) study (4), a diabetes prevention and management program for Native American youth. We assessed feasibility and acceptability of enrolling adult caregivers in the program and examined pilot data from caregivers enrolled in the TOD study to understand the preliminary effect of the program on these caregivers. Positive outcomes for youth enrolled in TOD were previously reported (5).

## Methods

We evaluated TOD, a 12-month home-visiting program delivered by Native American paraprofessional family health coaches to youth aged 10 to 19 years with or at-risk for diabetes, in a pre–post study conducted from November 2012 through July 2015 in partnership with 4 Native American reservation communities in the southwestern United States. Native American youth were referred to TOD by local Indian Health Services health care providers. Each youth identified a trusted adult aged 18 years or older living with or near them as their caregiver. Youth enrollment was not contingent on enrolling a caregiver. Informed consent was obtained from the youth (if the youth was a minor, permission was obtained from the parent or legal guardian and assent from the youth) and from the caregivers who agreed to participant in the study with the youth. The study was approved by relevant tribal review boards and the Johns Hopkins Bloomberg School of Public Health institutional review board.

The TOD curriculum consisted of 12 lessons for youth and 4 for caregivers. Caregivers were encouraged to attend all youth lessons. Caregivers and youth completed the demographic questionnaire at baseline. Satisfaction surveys were completed by caregivers and youth at 12 months. Self-report surveys were completed by caregivers and youth at baseline and at 3, 6, and 12 months (4,5). Physiologic data was collected at baseline and at 3, 6, and 12 months.

The study had a rolling enrollment for 20 months with 12 to 13 youth and caregivers enrolled each month. Caregivers’ physiologic data (weight, height, and waist measurements) were not originally part of the caregiver evaluation but were added at the request of caregivers three-quarters of the way through the study. Therefore, we had these measurements for only those caregivers who enrolled in the latter months of the study for baseline and 3, 6, and 12 months. Paired *t* tests, Fisher’s test, pairwise comparisons, and linear regression analysis were used to assess differences in dosage and characteristics of caregivers and youth across physiologic data availability and caregivers’ diabetes status. Paired *t* tests assessed caregiver physiologic changes between baseline (an average of data from baseline and 3 months) and at 12 months as evaluation time points. Linear regression analysis was conducted to examine the relationship between caregiver and youth weight loss.

## Results

A total of 256 youth enrolled in the study; of these, 226 (88%) enrolled with designated caregivers (4). We found no demographic differences between youth with and without a caregiver (unpublished data). A minority of caregivers (18.6%) reported that they had been diagnosed with diabetes. Caregiver age and screening history were the only demographic differences observed across caregiver diabetes status. Older caregivers were more likely to be diagnosed with diabetes, and those who were screened for diabetes were more likely to be diagnosed with diabetes then those who were not screened (Table 1).

**Table 1:** Demographic Characteristics of Youths (N = 256) and Their Caregivers (N = 226), Together on Diabetes (TOD) Study, by Physiologic Data Availability and Diabetes Status, 2012–2015^a^

Characteristic	Total	Has Physiologic Data^b^			Caregiver Diagnosed with Type 2 Diabetes?		
		No	Yes	*P*	No	Yes	*P*
**Youth**							
**Age, y**							
10–12	121 (47.3)	101 (46.1)	20 (54.1)	.08	90 (50)	21 (51.2)	.99
13–17	108 (42.2)	91 (41.6)	17 (45.9)		76 (42.2)	17 (41.5)	
>17	27 (10.6)	27 (12.3)	0 (0)		14 (7.8)	3 (7.3)	
**Male**	55.8 (143)	54.8 (120)	23 (62.2)	.40	106 (58.9)	16 (41.5)	.04
**Diabetes status**							
Has type 2 diabetes	29 (13.2)	25 (13.5)	4 (11.4)	.46	23 (14.7)	6 (17.1)	.80
Has prediabetes	111 (50.5)	90 (48.7)	21 (60)		81 (51.9)	13 (45.7)	
At risk for diabetes^c^	80 (36.3)	25 (37.8)	10 (28.6)		52 (33.3)	13 (37.1)	
**Caregiver**							
**Age, y**							
<35	68 (30.6)	55 (29.6)	13 (36.1)	.72	63 (35.8)	4 (9.8)	.01
35–44	97 (43.7)	83 (44.6)	14 (38.9)		72 (40.9)	23 (56.1)	
≥45	57 (25.7)	48 (25.8)	9 (25)		41 (23.3)	14 (34.2)	
**Male**	41 (18.3)	35 (18.6)	6 (16.7)	.78	35 (19.7)	5 (12.2)	.27
**Employed**	91 (40.6)	76 (40.4)	15 (41.7)	.90	72 (40.5)	16 (39)	.87
**Diabetes status**							
Screened for diabetes in past 3 months	48 (21.7)	37 (19.7)	11 (30.6)	.32	29 (16.3)	19 (46.3)	<.001
Has type 2 diabetes	41 (18.6)	34 (18.5)	7 (18.9)	.95	NA	NA	NA
**Is parent of participating youth**	171 (76.3)	145 (77.1)	26 (72.2)	.55	138 (77.5)	30 (73.1)	.21
**Feasibility (dosage)^d^**							
Number of caregiver lessons completed, mean (SD)	2.09 (1.62)	1.89 (.13)	3.06 (.23)	<.001	2.23 (.13)	1.68 (.28)	.07
Number of youth lessons attended by the caregiver, mean (SD)	4.71 (4.03)	4.18 (.29)	7.61 (.63)	<.001	5.73 (.32)	3.91 (.61)	.01
**Assessment of Together on Diabetes Program**							
Learned a lot	164 (95.9)	129 (96.3)	35 (94.6)	.65	126 (96.2)	34 (97.1)	.63
Would recommend TOD to others	167 (97.7)	130 (97)	37 (100)	.59	127 (97)	35 (100)	.58
Family health coach was helpful	168 (98.3)	131 (97.8)	37 (100)	.48	130 (99.2)	34 (97.1)	.38
Activities were helpful	168 (98.3)	131 (97.8)	37 (100)	.48	130 (99.2)	34 (97.1)	.38
Handouts were helpful	164 (95.9)	128 (95.5)	37 (100)	.34	128 (97.7)	32 (94.1)	.27
Referrals were helpful	157 (95.2)	123 (93.9)	37 (100)	.21	121 (95.3)	32 (97)	.56
**Number of visits were:**							
Too many	13 (7.7)	9 (6.8)	4 (10.8)	.35	12 (9.2)	1 (2.9)	.43
Just right	144 (85.2)	115 (87.1)	29 (78.4)		110 (84.6)	31 (88.5)	
Too few	12 (7.1)	8 (6.1)	4 (10.8)		8 (6.2)	3 (8.6)	
**Length of the program was:**							
Too long	5 (2.9)	4 (3)	1 (2.7)	.22	5 (3.9)	0 (0)	.72
Just right	149 (87.7)	119 (89.5)	30 (81.1)		113 (86.9)	32 (91.4 )	
Too short	16 (9.4)	10 (7.5)	6 (16.2)		12 (9.2)	3 (8.6)	
**Information taught by family health coaches was:**							
Too difficult	2 (2.4)	2 (1.5)	2 (5.4)	.23	4 (3.1)	0 (0)	.10
Just right	162 (95.3)	127 (95.5)	35 (94.6)		125 (96.2)	33 (94.8)	
Too easy	2 (2.4)	4 (3.1)	0 (0)		1 (.8)	2 (5.7 )	

Abbreviation: NA, not applicable; SD, standard deviation.

a Values are n (%) unless otherwise indicated.

b Physiologic data including height, weight, and waist circumference were collected on a subset of caregivers. The collection of physiologic data was not part of the original evaluation and was added three-quarters of the way through the study. The study had rolling enrollment (~12-13 caregivers enrolled over 20 months). Therefore, only those enrolled in the latter months of study enrollment had time point 1 month and 12 months physiologic data collected.

c The criteria used to define youth at risk for diabetes were determined by physicians who were practicing in the 4 program sites. Youth at risk had a body mass index z-score greater than the 85th percentile for age and sex and at least one of the following qualifying laboratory test results: low-density lipoprotein at or higher than100 mg/dL (2.6 mmol/L), triglycerides at or higher than 150 mg/dL (1.7 mmol/L), or high-density lipoprotein at or less than 40 mg/dL (1 mmol/L).

d The number of lessons attended by the caregiver.

**Feasibility and acceptability**. Caregivers’ lesson completion and attendance at youth lessons was moderate (Table 1). Caregivers of youth with diabetes attended significantly more youth lessons than caregivers of youth at risk for diabetes (6.5 vs 3.79; *P* = .01). Additionally, youth age and number of youth lessons were positively correlated, the younger the youth, the more lessons the caregiver attended (*R*2 = .0193; *P* = .04). Neither youth age or diabetes status was associated with the number of lessons caregivers completed. However, caregivers who did not have diabetes attended more youth lessons (*P* = .01) (Table 1). Caregiver satisfaction was high (Table 1). Through open-ended questions, caregivers reported that they liked the knowledge they gained through the TOD program and TOD program activities and liked that the family health coach came to their home. Some said they did not like the program’s time commitment.

**Program impact.** Only 37 (16.4%) caregivers had physiologic data; this was primarily due to the late addition of the collection of caregiver physiologic measures because no differences in demographic variables were observed across caregivers by physiologic data availability. The caregivers for whom we had physical measurements attended more youth lessons than those without (Table 1), lost a significant amount of body weight (mean, 5.9 lb), and had a reduction in waist circumference (mean = 1.66 cm) (Table 2). Changes in caregiver body mass index (body weight in kilograms divided by height in m^2^) and youth body mass index z-score (measures of relative weight adjusted for youth age and sex) from baseline to 12 months were not related (*P* = .93).

**Table 2:** Program Impact on a Subset of Caregivers (N = 37), Together on Diabetes (TOD) Study, 2012–2015^a^

Physiologic Changes, Mean (Standard Deviation)^b^	Time point 1^c^	12 Months	*P* Value
Weight, lb (N = 37)	216.4 (8.77)	210.5 (8.72)	.004
Body mass index (N = 35)	38.12 (1.57)	37.28 (1.53)	.02
Waist circumference, cm (N = 35)	122.14 (3.06)	120.48 (3.04)	.15

a Physiologic data at time points 1 month and 12 months were available from only 37 caregivers. The collection of physiologic data was not part of the original evaluation and was added three-quarters of the way through the study. The study had rolling enrollment (~12-13 caregivers enrolled over 20 months). Therefore, only those enrolled in the latter months of study enrollment had physiologic data collected for time points 1 month and 12 months.

b A total of 37 caregivers had weight collected, but height and waist circumference were collected from only 35 caregivers.

c Time point 1= average between baseline and 3-month assessment.

## Discussion

Three-quarters of the way through the TOD trial, we suspected caregivers were making lifestyle changes along with the youth they were sponsoring. Parents often shared that their youths motivated them to be healthier caregivers. Thus, in addition to feasibility, acceptability, and demographic data on caregivers that we collected from the beginning of the study, we added collection of physical measurements to the caregiver evaluation. Although physiologic data were available on less than 20% of caregivers, dosage, satisfaction, and demographic data were available for all. Pilot results presented here build on and support previous research indicating family engagement is feasible and beneficial in diabetes prevention efforts among Native American (6). However, results indicate that program developers should make more of an effort to engage and track the progress of the caregivers in TOD and other programs. Because program satisfaction was reported as high, caregiver participation in fewer than half of lessons may have been due to lack of emphasis on their involvement by the program developers and managers and work-related conflicts and not because caregivers did not want to be involved. Furthermore, the role of youth age and youth and caregiver diabetes status in caregiver engagement should be explored in future studies, because it appears that the TOD program was more successful at engaging caregivers of younger youths (≤15 y), caregivers of youth with diabetes, and caregivers who did not have diabetes themselves.

We were able to tell that caregivers for whom we had data on physical measures significantly reduced their weight despite TOD’s sole focus on youth goals. Because this is pilot data, results should be interpreted with caution. The correlation between youth and caregiver weight loss documented in past research (7) was not observed in our study. This could be attributed to our small sample size. Nonetheless, results indicate that future diabetes prevention programs in Native American communities should engage and collect parallel behavioral and physiological data from youth–adult dyads and, ideally, from other family members.

Ours is one of the first studies to examine the feasibility, acceptability, and preliminary effect of engaging adult caregivers in youth diabetes prevention programs. Our findings coupled with previous research support intergenerational, family-based diabetes prevention programs in Native American communities.
